# I Want to (Bud) Break Free: The Potential Role of *DAM* and *SVP*-Like Genes in Regulating Dormancy Cycle in Temperate Fruit Trees

**DOI:** 10.3389/fpls.2018.01990

**Published:** 2019-01-10

**Authors:** Vítor da Silveira Falavigna, Baptiste Guitton, Evelyne Costes, Fernando Andrés

**Affiliations:** Amélioration Génétique et Adaptation des Plantes Méditerranéennes et Tropicales, Université de Montpellier, Centre de Coopération Internationale en Recherche Agronomique pour le Développement, Institut National de la Recherche Agronomique, Montpellier SupAgro, Montpellier, France

**Keywords:** phenology, bud dormancy, *SVP*-like genes, bud break, seasonal, temperate tree species, MADS-box family transcription factors

## Abstract

Bud dormancy is an adaptive process that allows trees to survive the hard environmental conditions that they experience during the winter of temperate climates. Dormancy is characterized by the reduction in meristematic activity and the absence of visible growth. A prolonged exposure to cold temperatures is required to allow the bud resuming growth in response to warm temperatures. In fruit tree species, the dormancy cycle is believed to be regulated by a group of genes encoding MADS-box transcription factors. These genes are called *DORMANCY-ASSOCIATED MADS-BOX* (*DAM*) and are phylogenetically related to the *Arabidopsis thaliana* floral regulators *SHORT VEGETATIVE PHASE* (*SVP*) and *AGAMOUS-LIKE 24*. The interest in *DAM* and other orthologs of *SVP* (*SVP*-like) genes has notably increased due to the publication of several reports suggesting their role in the control of bud dormancy in numerous fruit species, including apple, pear, peach, Japanese apricot, and kiwifruit among others. In this review, we briefly describe the physiological bases of the dormancy cycle and how it is genetically regulated, with a particular emphasis on *DAM* and *SVP*-like genes. We also provide a detailed report of the most recent advances about the transcriptional regulation of these genes by seasonal cues, epigenetics and plant hormones. From this information, we propose a tentative classification of *DAM* and *SVP*-like genes based on their seasonal pattern of expression. Furthermore, we discuss the potential biological role of *DAM* and *SVP*-like genes in bud dormancy in antagonizing the function of *FLOWERING LOCUS T*-like genes. Finally, we draw a global picture of the possible role of *DAM* and *SVP*-like genes in the bud dormancy cycle and propose a model that integrates these genes in a molecular network of dormancy cycle regulation in temperate fruit trees.

## Description of Biological Phenomenon During Dormancy Cycle

**FIGURE 1:**
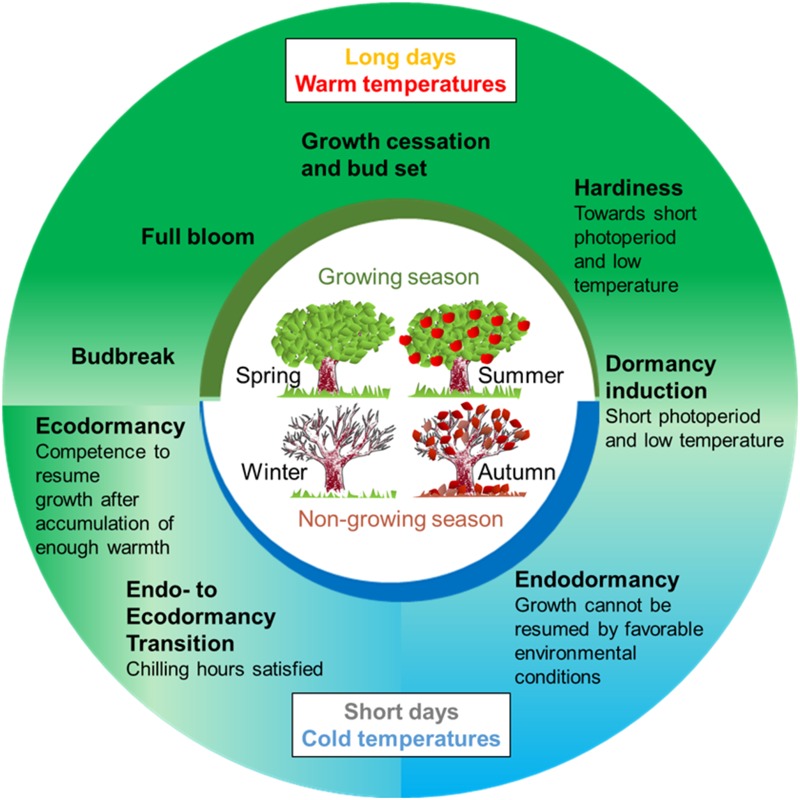
Representation of 1-year life cycle of a temperate fruit tree. Trees grow actively during the growing season, typically in spring and early summer. At the end of summer and beginning of autumn, they initiate growth cessation, presumably in response to short days. Toward autumn, trees increase their resistance to cold (hardiness) and buds enter in dormancy during autumn. Chilling temperatures during the winter periods triggers dormancy release. Then, ecodormant buds can respond to warm temperatures in the spring to promote budbreak, which is followed by active growth at the end of the spring and in the summer.

Temperate trees are distributed over a geographical zone of the globe that spans between the tropics and the polar regions. These regions present wide temperature ranges and seasonal changes all over the year. To survive these conditions, trees adjust their annual growth cycle to the seasonal environmental changes: they grow during the favorable seasons and progressively stop their growing activity, until the growth cessation of all meristems during the unfavorable ones (Figure 1). This plasticity is possible thanks to mechanisms of environment perception (i.e., day-length and temperature) and signaling pathways that are integrated in developmental programs. One crucial developmental program that allows adaptation to the low temperatures of winter is dormancy. In many of these species, dormancy is induced by the shortening of the day length that preludes the advent of the winter (Kramer, 1936). Dormancy can be defined as “a state of self-arrest of the shoot apical meristem (SAM) which is maintained under growth-promoting conditions” (Paul et al., 2014). However, it must be noted that dormancy also concerns buds that do not contain meristematic tissues any more, such as floral buds in *Prunus* species that contain a single flower. Lang et al. (1987) proposed that dormancy in temperate trees can be divided in three phases: paradormancy, endodormancy and ecodormancy. Paradormancy, also known as inhibition by correlation (Champagnat, 1989; Crabbe and Barnola, 1996), consists in the inhibition of growth regulated by hormones and competition among organs. In this stage, buds are competent to grow if separated from other parts of the plant. At the end of the autumn, the reduction of the photoperiod and the exposure to low temperatures induce growth cessation in all SAM and the formation of winter buds that protect the meristematic tissues, and thereafter endodormancy. During endodormancy, bud growth is inhibited by internal signals, and it is only overcome by a period of chilling temperatures (Lang et al., 1987; Anderson, 2015). It is associated with the mobilization of sugars and acquisition of cold hardiness. The amount of cold needed to release endodormancy, usually referred to as chilling requirement (CR), is species- and cultivar-dependent suggesting a strong genetic control of the trait (Olukolu et al., 2009; Falavigna et al., 2015). Once endodormancy is released, buds enter into ecodormancy during which they are competent to resume growth if experiencing warm conditions (Figure 1). However, bud growth’s reactivation is inhibited as long as environmental conditions remain temporary unfavorable (e.g., cold temperatures) and is initiated after a period of increasing temperatures and after a sufficient amount of heat (referred as heat requirement, HR).

Environmental conditions (e.g., temperature and photoperiod) are perceived by plants through complex molecular networks and endogenous signals (e.g., plant hormones, oxidative stresses and energy metabolism) that regulate growth and dormancy cycle (i.e., growth cessation, dormancy phases and budbreak) in the SAM and more generally in buds. These networks have been studied in detail in SAM of some tree species, such as birch and poplar (see Singh et al., 2017). In response to short days, the symplasmic intracellular communication in the SAM is blocked by the deposition of callose (1,3-β-glucan) in the plasmodesmata. It is believed that the blockage of the SAM symplasmic paths restricts the transport of growth regulators, including orthologs of the Arabidopsis [*Arabidopsis thaliana* (L.) Heynh] FLOWERING LOCUS T (FT) protein (Rinne et al., 2011; Andrés and Coupland, 2012; Tylewicz et al., 2018), leading to growth arrest and eventually to dormancy (Rinne and van der Schoot, 1998; Rinne and Kaikuranta, 2001). Recently, it was shown that the plant hormone abscisic acid (ABA) accumulates in response to short days in the SAM and contributes to plasmodesmata closure (Tylewicz et al., 2018). However, whether ABA influences this process by directly affecting callose deposition remains unclear. As mentioned above, endodormancy can be overcome by period of exposure to low temperature. Indeed, such temperature promotes the expression of genes encoding a subset of glucan hydrolase 17 (GH17) family members. The expression of these genes is correlated with the removal of the callose deposition from the plasmodesmata, and the consequent reactivation of the symplastic connectivity and the SAM growth (probably by allowing the transport of FT, among other growth-promoting substances, to the SAM) (Rinne et al., 2011). Interestingly, the exogenous application of the plant hormone gibberellin (GA) can replace low temperatures in dormancy release and induce the expression of specific *GH17* genes (Rinne et al., 2011), suggesting a role of GA in this process. Other signals may be also involved in dormancy release and budbreak. In grapevine, the meristem isolation during dormancy triggers a series of hypoxia responses, including starch hydrolysis (Rubio et al., 2014) and signaling cascades (Meitha et al., 2015, 2018), that ultimate in dormancy release and budbreak.

Compared to birch and poplar, our knowledge about dormancy cycle regulation by molecular mechanisms is much more limited in temperate fruit species. In these species temperature is the major factor affecting dormancy release and budbreak (Cook and Jacobs, 1999; Heide and Prestrud, 2005; Guo et al., 2014; Li et al., 2016) and therefore, they are extremely vulnerable to global warming. Temperature influences the tree phenology in the orchards by affecting winter cold fulfillment and the timing of growth resumption after dormancy. Perturbations of the dormancy cycle are already visible in changes of the time of budbreak, flowering synchronization between cultivars with consequences regarding reduction of the yield (Luedeling et al., 2011; Legave et al., 2013, 2015; Guo et al., 2015). However, the negative consequences for fruit tree production could be reduced or even nullified by adapting the CR of varieties to temperature changes at regional scale. Thus, the understanding of the dormancy cycle, its relationship to environmental factors and its molecular control is crucial to characterize existing cultivars and to obtain new ones better adapted to future scenarios of temperature increase. For this reason, an important effort on the understanding of the genetic and molecular control of dormancy cycle has been made during the last years.

In this review, we focus in the current knowledge of the molecular control of dormancy cycle mainly (but not solely) in temperate fruit tree species. Specifically, we focus on a group of genes that recently emerged as potential regulators of the dormancy cycle in these tree species. These genes were first identified more than 10 years ago and named as *DORMANCY-ASSOCIATED MADS-BOX* (*DAM*) genes (Bielenberg et al., 2008; Horvath et al., 2008). They were found phylogenetically related to the Arabidopsis floral regulators *SHORT VEGETATIVE PHASE* (*SVP*) and *AGAMOUS-LIKE 24* (*AGL24*). In what follows, we discuss the discovery of *DAM* genes, their most important structural and evolutionary features, how they are transcriptionally regulated, and how they might control the dormancy cycle. For obtaining a more global picture of dormancy regulation, the readers could also refer, among others, to any of these previous reviews: Rohde and Bhalerao (2007), Campoy et al. (2011), Cooke et al. (2012), Considine and Foyer (2014), Shim et al. (2014), Paul et al. (2014), Maurya and Bhalerao (2017), and Beauvieux et al. (2018).

## The Genetic Determinism of Bud Dormancy is Associated With the *DAM* Genes

The study of the bud dormancy cycle gained a huge attention in the last two decades especially due to the agronomical disorders caused by global warming. New genetic and molecular tools were combined to the employment of natural mutants, contrasting cultivars and observation of phenological stages, rendering the first advances in the field. One of the most emblematic studies made use of the peach [*Prunus persica* (L.) Batsch] mutant called *evergrowing* (*evg*), which fails to form terminal vegetative buds and maintains constant growth in response to dormancy-inducing conditions. The *evg* trait is genetically heritable and segregates as a single recessive gene (Rodriguez-A et al., 1994). The *evg* locus was tracked to peach linkage group 1 (LG1), and further mapping and sequencing of the locus revealed a genomic deletion affecting six tandemly repeated MIKC^c^-type MADS-box genes, which were named *PpeDAM1* to *PpeDAM6* (Bielenberg et al., 2008). While *PpeDAM1* to *PpeDAM4* were physically deleted, the expression levels of *PpeDAM5* and *PpeDAM6* were reduced in the *evg* mutant (Bielenberg et al., 2008). Comparisons to the model plant Arabidopsis showed that *DAM* genes share sequence homology to *SVP*, and are sometimes referred to as *SVP*-like genes. Additional genetic studies in peach identified several minor quantitative trait locus (QTL) associated with bud dormancy, but the most significant QTL for CR usually overlapped with the *evg* locus (Fan et al., 2010; Romeu et al., 2014; Zhebentyayeva et al., 2014; Bielenberg et al., 2015). These results suggested that the *DAM* genes might be one of the most relevant genetic elements underlying CR in peach. In Japanese apricot (*Prunus mume* Sieb. et Zucc.), genomic library screening and shotgun sequencing revealed a peach-like genome structure, with six homologs of *PpeDAM* genes tandemly arrayed (Sasaki et al., 2011). Moreover, genetic analyses for CR, HR and *PmuDAM6* expression identified a QTL in LG4, suggesting that this locus may control dormancy release, budbreak and *PmuDAM6* downregulation in Japanese apricot leaf buds (Kitamura et al., 2018). In other *Prunus* species, the genetic determinisms of CR and flowering time were revealed, with QTLs coinciding with the location of *DAM* genes in almond [*Prunus dulcis* (Miller) D. A. Webb], apricot (*Prunus armeniaca* L.) and sweet cherry (*Prunus avium* L.) (Olukolu et al., 2009; Sánchez-Pérez et al., 2012; Castède et al., 2015). However, the most significant QTLs for these species were found in other loci, highlighting the complex genetic control of these characters. These findings suggest that *DAM* genes may be involved in the control of CR and flowering time in other *Prunus* species besides peach, although other genes possibly involved still have to be unveiled.

Genetic studies in apple (*Malus* x *domestica* Borkh.) and pear (*Pyrus communis* L.) also identified QTLs for budbreak and flowering time overlapping with *DAM* genes. In apple, the employment of a multifamily and pedigree-based analysis revealed QTLs that co-localize with *DAM* genes in LG8 and LG15 (Allard et al., 2016), although the most recurrent QTL in apple is in LG9 (van Dyk et al., 2010; Celton et al., 2011; Urrestarazu et al., 2017). The QTLs in LG9 and LG8 were also identified in pear, consistently with the high synteny between the species (Gabay et al., 2017). Indeed, the genomes of *Malus* and *Pyrus* are highly syntenic, especially because they underwent a recent whole-genome duplication (WGD) event that is not shared with other Rosaceae clades such as the one that *Prunus* belongs to (Xiang et al., 2017). In apple, the genomic composition of *DAM* genes were identified, but the number of detected *DAM* genes was not consistent among reports (Mimida et al., 2015; Wisniewski et al., 2015; Kumar et al., 2016). Porto et al. (2016) proposed a unified nomenclature composed of four apple *DAM* genes (*MdoDAM1* to *MdoDAM4*) and two *SVP*/*JOINTLESS* (*J*)-like genes, although not all genes previously named as *DAM* were considered. A recent study functionally characterized *MdoDAMb* (Wu et al., 2017a), a gene that was excluded from this unified nomenclature due to bad gene prediction in the first apple genome version (Velasco et al., 2010). In Japanese pear (*Pyrus pyrifolia* Nakai), three *DAM* genes (called *PpyMADS13* by the authors) were identified by PCR amplification using primers based on the sequence of *PpeDAM6* (Ubi et al., 2010; Saito et al., 2013). By exploring the availability of the Chinese white pear (*Pyrus bretschneideri* Rehd.) genome, three *DAM* genes were identified and named *PpyDAM1*-*3* (Niu et al., 2016). In both apple and pear, *DAM* genes have close chromosomal locations and are present in the syntenic chromosomes 8 and 15. Interestingly, apple chromosome 8 is highly syntenic to the end of the peach chromosome 1 (Porto et al., 2016), where *PpeDAM* genes are located. This suggests that the recent WGD event shared by apples and pears generated two tandem regions containing *DAM* genes in these species. Tandem regions *per se* are difficult to map and annotate, and this partially explains why different studies found distinct quantities of *DAM* members in these species.

Besides Rosaceous species, *DAM* and *SVP*-like genes were also identified and related to dormancy cycle in other species. In the herbaceous perennial weed leafy spurge (*Euphorbia esula* L.), two *EesDAM* transcripts were identified (Horvath et al., 2008, 2010), but further characterization demonstrated that these transcripts result from alternative splicing of a single *EesDAM* gene (Horvath et al., 2013). Four genes similar to *SVP* were identified in the perennial kiwifruit vine (*Actinidia* spp.) (Wu et al., 2012). Recently, a gene called *SVL* (*SHORT VEGETATIVE PHASE-LIKE*) was identified in hybrid aspen (*Populus tremula* L. × *P*. *tremuloides* Michx.) and related to the regulation of budbreak (Singh et al., 2018).

## Structure and Phylogeny of DAM and SVP-Like Proteins

**FIGURE 2:**
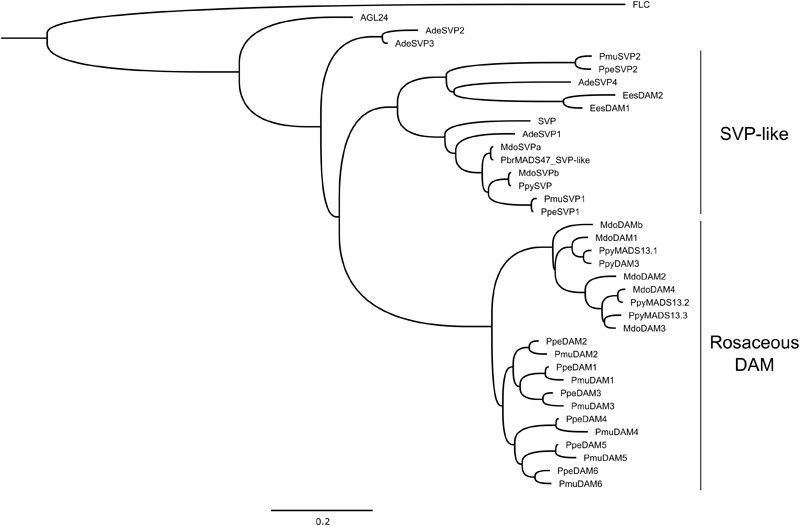
Phylogenetic analysis of DAM and SVP-like proteins from several plant species (Ade, *Actinidia deliciosa*; Ees, *Euphorbia esula*; Mdo, *Malus* × *domestica*; Pmu, *Prunus mume*; Ppe, *Prunus persica*; Pbr, *Pyrus bretschneideri*; Ppy, *Pyrus pyrifolia*). Arabidopsis AGL24, FLC and SVP were also used in the analysis. Sequences were aligned using MAFFT (Katoh et al., 2002). Poorly aligned positions and divergent regions were eliminated using Gblocks (Castresana, 2000). Maximum likelihood phylogeny was inferred using PhyML 3.0 (Guindon and Gascuel, 2003). The tree was drawn using FigTree v1.4.3 (http://tree.bio.ed.ac.uk/software/figtree/). Arabidopsis FLC was used to root the tree. The scale bar unit represents branch lengths (0.2 substitutions/site). Accession codes (NCBI): AGL24 (AT4G24540), FLC (AT5G10140), SVP (AT2G22450), AdeSVP1 (AFA37963), AdeSVP2 (AFA37964), AdeSVP3 (AFA37965), AdeSVP4 (AFA37966), EesDAM1 (ABY53594), EesDAM2 (ABY60423), MdoDAM1 (AOA32865), MdoDAM2 (AOA32866), MdoDAM3 (XP_017186028), MdoDAM4 (AOA32868), MdoDAMb (ADL36743), MdoSVPa (AOA32867), MdoSVPb (BAR40332), PbrMADS47 (XP_009364259), PmuDAM1 (BAK78921), PmuDAM2 (BAK78922), PmuDAM3 (BAK78923), PmuDAM4 (BAK78924), PmuDAM5 (BAK78920), PmuDAM6 (BAH22477), PmuSVP1 (AML81015), PmuSVP2 (AML81016), PpeDAM1 (ABJ96361), PpeDAM2 (ABJ96363), PpeDAM3 (ABJ96364), PpeDAM4 (ABJ96358), PpeDAM5 (ABJ96359), PpeDAM6 (ABJ96360), PpeSVP1 (XP_020422316), PpeSVP2 (XP_020409383), PpyMADS13.1 (BAI48074), PpyMADS13.2 (BAI48075), PpyMADS13.3 (BAM74166), PpyDAM3 (AJW29049), PpySVP (AJW29050).

The DAM proteins belong to the plant exclusive type II MADS-box transcription factors called MIKC^c^, which present a characteristic arrangement of four major domains (Figure 2). The MADS-box domain is involved in DNA binding, while the I and K domains are essential for protein dimerization and higher-order complex formations (Kaufmann et al., 2005). The role of the C region is less clear and may be involved in protein complex formation and transcriptional regulation (Smaczniak et al., 2012). The MIKC^c^ transcription factors are divided into 13 subfamilies originated from ancestral seed plants (Smaczniak et al., 2012). Phylogenetic and molecular evolution analysis of the *DAM* genes classified them as belonging to the StuMADS11/AGL24/SVP subfamily of MADS-box transcription factors (Jiménez et al., 2009).

A new simplified phylogeny is proposed for DAM and SVP-like proteins (Figure 2), in order to represent previously published data, as well as to update the information based on recent findings. For apple DAMs, we followed the nomenclature proposed by Porto et al. (2016), while for Japanese pear we followed the nomenclature proposed by Ubi et al. (2010) and Saito et al. (2013), with an additional DAM identified by Niu et al. (2016) referred as PpyDAM3. The proteins grouped into two major clusters (Figure 2). All Rosaceous DAM proteins formed a cluster subdivided in two groups; one containing *Prunus* DAM proteins and other from *Malus* and *Pyrus* (Figure 2). This highlights the evolutionary similarities of these Rosaceous genera, but at the same time the differences among genus. Another well-defined cluster was composed of SVP-like proteins of Arabidopsis, apple, Japanese apricot, kiwifruit, peach, and Japanese pear, together with leafy spurge DAM proteins. The segregation of SVP-like proteins in a different cluster suggests an evolutionary diversification among *DAM* and *SVP*-like genes within Rosaceae. This could be interpreted as a process of neofunctionalization between *DAM* and *SVP*-like genes, and subfunctionalization within the *DAM* genes. The latter has been proposed for *PpeDAM* genes in peach (Jiménez et al., 2009; Li et al., 2009).

## Expression Patterns and Environmental Control of *DAM* and *SVP*-Like Genes

**FIGURE 3:**
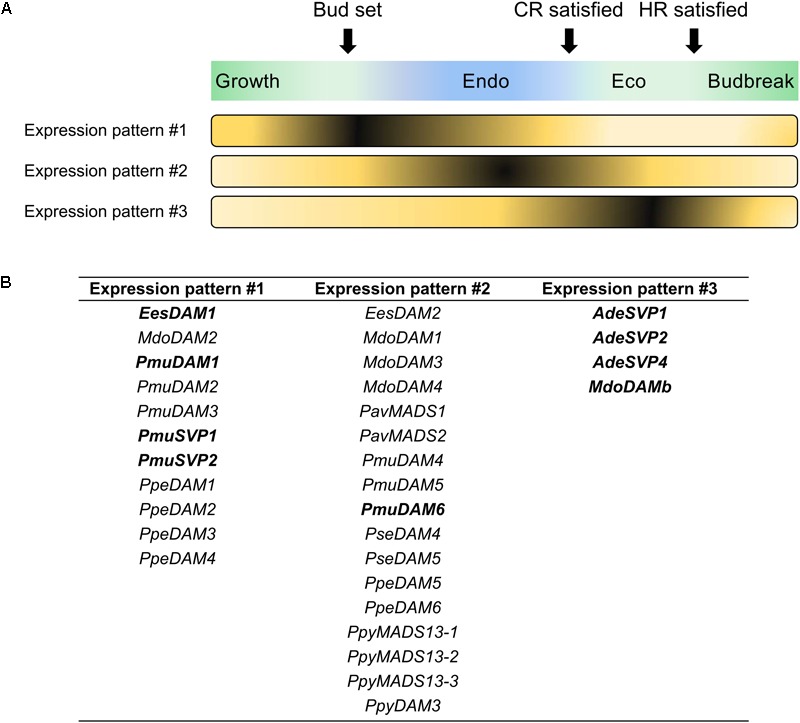
Tentative consensus of seasonal *DAM* and *SVP*-like gene expression dynamics. Data were gathered from studies marked with an asterisk in Table 1. Three different expression patterns **(A)** were identified for *DAM* and *SVP*-like genes **(B)**. Additional information concerning the different dormancy cycle phases are present in Figure 1. *AdeSVP3, MdoSVPa, MdoSVPb* and *PpySVP* did not present a consistent seasonal transcript variation. Genes in bold were functionally characterized (see the sections “Functional Studies of *DAM* and *SVP*-Like Genes of Fruit Tree Species in Model Plants” and “*DAM* and *SVP*-Like Genes Might Act as Growth Inhibitors in Trees”).

The quantification of *DAM* transcript levels during the year has shown that their mRNA expression profiles correlate to different dormancy cycle phases. A summary of the transcriptomic studies that identified *DAM* genes differentially expressed during bud dormancy is presented in Table 1. Despite the employment of different quantification techniques and plant materials (e.g., apical or lateral buds, flower or vegetative buds, etc.), the *DAM* genes remarkably presented seasonal expression patterns along the year. Interestingly, these profiles were somehow consistent even among different species. Here, we clustered the expression patterns of *DAM* genes from several species (i.e., apple, Japanese pear, Japanese apricot, peach, sweet cherry, Chinese cherry, leafy spurge and kiwifruit) into three different groups based on their seasonal pattern of expression during the growth and dormancy cycle (Figure 3). Some Rosaceous genes do not show a consistent pattern of expression among different publications and thus, they are not included in Figure 3. Additionally, we excluded *DAM* and *SVP*-like genes from poplar (Howe et al., 2015) and tea plant (Hao et al., 2017), as their seasonal expression pattern did not fit in any of the proposed groups. Whether this indicates that these genes are not involved in dormancy cycle or have a different role in poplar and tea plant remains uncertain. We attempted to assign a function to the genes belonging to each group depending on the particular dormancy cycle phase during which their expression was maximal. This function was assigned based on the proposed role for a subset of genes of a given group that were already functionally characterized (see the section “What Do We Know About the Biological Function of *DAM* and *SVP*-Like Genes?”) and might not reflect the precise function of all the genes of the group. However, for many of the genes included in Figure 3, the expression profile is the only information present in the literature that could help us to infer their biological function.

**Table 1:** Summary of transcriptome studies during dormancy, highlighting the ones that identified *DAM* and *SVP*-like genes.

Species	Technique	Strategy	*DAM* genes	Reference
Peach (*Prunus persica*)	Northern blot	*evg* mutant	*PpeDAM1*-*6*	Bielenberg et al., 2008^∗^
	Real-time PCR	Contrasting CR cultivars and controlled cold exposure	*PpeDAM5*-*6*	Jiménez et al., 2010b^∗^
	Real-time PCR	Contrasting CR cultivars, controlled cold exposure and cyanamide treatment	*PpeDAM5*-*6*	Yamane et al., 2011a^∗^
	Real-time PCR	Contrasting CR cultivars and controlled cold exposure	*PpeDAM5*-*6*	Yamane et al., 2011b^∗^
	Real-time PCR	Contrasting CR cultivars	*PpeDAM5*-*6*	Yamane et al., 2011c^∗^
	Real-time PCR	Contrasting CR cultivars	*PpeDAM4*-*6*	Leida et al., 2012^∗^
	Semi-quantitative PCR	Annual growth cycle	*PpeDAM1*-*6*	Li et al., 2009^∗^
	SSH	WT vs. dormancy-incapable mutant (*evg*)	*PpeDAM1, PpeDAM6*	Jiménez et al., 2010a
	Suppression subtractive hybridization	Contrasting CR cultivars	*PpeDAM4*-*6*	Leida et al., 2010^∗^
Japanese apricot (*Prunus mume*)	Microarray	Dormancy cycle	*PmuDAM1, PmuDAM3*-*6*	Habu et al., 2014^∗^
	Real-time PCR	Contrasting CR cultivars and controlled cold exposure	*PmuDAM1*-*6*	Sasaki et al., 2011^∗^
	Real-time PCR	Dormancy cycle	*PmuDAM1*-*6*	Zhao et al., 2018a^∗^
	Real-time PCR	Dormancy cycle	*PmuDAM6*	Zhao et al., 2018b^∗^
	Real-time PCR	Annual growth cycle	*PmuDAM1*-*6*	Zhao et al., 2018c
	Real-time PCR	Dormancy cycle	*PmuSVP1, PmuSVP2*	Li et al., 2017^∗^
	RNA-seq	Dormancy cycle	*PmuDAM4*-*6*	Habu et al., 2012
	RNA-seq	Dormancy cycle	*PmuDAM3, PmuDAM5*-*6*	Zhong et al., 2013
	RNA-seq	Dormancy cycle	*PmuDAM1*-*6*	Zhang et al., 2018
	SSH	Dormancy cycle	*PmuDAM6*	Yamane et al., 2008^∗^
Apple (*Malus* × *domestica*)	Real-time PCR	Annual growth cycle	*MdoDAM1*-*2*	Wisniewski et al., 2015^∗^
	Real-time PCR	Dormancy cycle	*MdoDAM1*-*2*	Mimida et al., 2015^∗^
	Real-time PCR	Contrasting CR cultivars and controlled cold exposure	*MdoDAM1*-*4*	Porto et al., 2016^∗^
	Real-time PCR	Dormancy cycle	*MdoDAM1*-*2, MdDAMb*	Wu et al., 2017a^∗^
	RNA-seq	Dormancy cycle	*MdoDAM1, MdoDAM3*	Kumar et al., 2016^∗^
	RNA-seq	Contrasting chilling availability	*MdoDAM1*-*3*	Kumar et al., 2017
	SSH	Contrasting CR cultivars	*MdoDAM1*	Falavigna et al., 2014^∗^
	Real-time PCR	Dormancy cycle, controlled heat exposure and cyanamide treatment	*PpyMADS13-1, PpyMADS13-2, PpyMADS13-3*	Saito et al., 2013^∗^
	Real-time PCR	Dormancy cycle	*PpyMADS13-1*	Saito et al., 2015^∗^
	Real-time PCR	Controlled cold and heat exposure	*PpyMADS13-1, PpyMADS13-2, PpyMADS13-3*	Ito et al., 2016
	Real-time PCR	Dormancy cycle	*PpyDAM1*-*3, PpySVP*	Niu et al., 2016^∗^
	Real-time PCR	Dormancy cycle and cyanamide treatment	*PpyMADS13-1*	Tuan et al., 2017^∗^
	RNA-seq	Dormancy cycle	*PpyMADS13-1, PpyMADS13-2*	Liu et al., 2012^∗^
	RNA-seq	Dormancy cycle	*PpyMADS13-1, PpyMADS13-2, PpyMADS13-3*	Bai et al., 2013^∗^
Kiwifruit (*Actinidia* spp)	Real-time PCR	Annual growth cycle and cyanamide treatment	*AdeSVP1*-*4*	Wu et al., 2012^∗^
Chinese cherry (*Prunus pseudocerasus*)	RNA-seq	Dormancy cycle	*PpcDAM3*-*6*	Zhu et al., 2015^∗^
Sweet cherry (*Prunus avium*)	Real-time PCR	Dormancy cycle	*PavMADS1*-*2*	Rothkegel et al., 2017^∗^
Raspberry (*Rubus idaeus*)	Microarray	Dormancy cycle	*SVP*-like/*DAM*-like	Mazzitelli et al., 2007
Leafy spurge (*Euphorbia esula*)	Microarray	Dormancy cycle	*EesDAM1*-*2*	Horvath et al., 2008^∗^
	Northern blot	Dormancy cycle	*EesDAM1*-*2*	Horvath et al., 2010^∗^
	Northern blot	Dormancy cycle	*EesDAM1*-*2*	Horvath et al., 2013^∗^
	Real-time PCR	Dormancy cycle	*EesDAM1*-*2*	Doğramaci et al., 2010^∗^
	Real-time PCR	Dormancy cycle	*EesDAM1*-*2*	Hao et al., 2015^∗^
Poplar (*Populus trichocarpa*)	Microarray	Dormancy cycle	*DAM*-like	Howe et al., 2015
Tea (*Camellia sinensis*)	RNA-seq	Dormancy cycle	*DAM*-like	Hao et al., 2017

*^∗^Studies used to build Figure 3. SSH, suppression subtractive hybridization.*

*DAM* genes showing the expression pattern #1 presented a peak of expression (approximately) during bud set, i.e., in the transition from summer to autumn (Figure 3). Peach genes displaying this pattern were proposed to have a role in the regulation of seasonal growth cessation and terminal bud formation (Li et al., 2009). As *DAM* genes from Japanese apricot (Sasaki et al., 2011; Zhao et al., 2018a), apple (Mimida et al., 2015; Wisniewski et al., 2015; Porto et al., 2016; Wu et al., 2017a) and leafy spurge (Horvath et al., 2008, 2013) showed a similar expression pattern, we can hypothesize that some of these genes may share the same role as the peach genes. A wide range of sampling materials were used to quantify these genes, such as terminal tissues (peach), lateral vegetative and floral buds (Japanese apricot), crown buds (leafy spurge), or apical buds and bark tissues (apple).

The vast majority of *DAM* genes showed the expression pattern #2, presenting their highest expression levels during endodormancy. Their subsequent transcriptional downregulation was correlated with the satisfaction of CR or HR. This pattern of expression is compatible with a role as quantitative repressors of endodormancy release and/or budbreak, as suggested for *PmuDAM6* (Sasaki et al., 2011). The expression pattern #2 was identified for *DAM* genes of Japanese apricot (Sasaki et al., 2011; Zhao et al., 2018a,b), peach (Li et al., 2009; Jiménez et al., 2010b; Yamane et al., 2011a,b,c), Japanese pear (Ubi et al., 2010; Liu et al., 2012; Bai et al., 2013; Saito et al., 2013; Ito et al., 2016; Niu et al., 2016), apple (Falavigna et al., 2014; Mimida et al., 2015; Wisniewski et al., 2015; Kumar et al., 2016, 2017; Porto et al., 2016; Wu et al., 2017a), sweet and Chinese cherry (Zhu et al., 2015; Rothkegel et al., 2017), and leafy spurge (Horvath et al., 2008, 2013). Again, a wide range of tissues were employed in the quantification of these genes.

*PpeDAM1/PpeDAM4* (expression pattern #1) and *PpeDAM5*/*PpeDAM6* (expression pattern #2) are expressed when the day-length is short and the temperature is low. Interestingly, these genes were up-regulated in experiments where peach plants were transferred from long-days (16/8 h light/dark) to short-days (8/16 h light/dark) in controlled environments (Li et al., 2009). To know if these genes are also induced by cold, peach branches sampled in the autumn were maintained in a constant 12 h light/dark photoperiod cycle and exposed to two temperature regimes: around 25°C or around 15°C. The expression of *PpeDAM5* and *PpeDAM6* was up-regulated in lateral vegetative buds by the 15°C treatment (Yamane et al., 2011a). These experiments suggested that both short-days conditions and short-term low temperature exposure trigger the expression of these *DAM* genes. From these studies, it can be hypothesized that each *DAM* gene could integrate distinct environmental signals (such as photoperiod and/or temperature) in order to allow a fine-tuning regulation of the different phases of tree dormancy cycle, with genes showing expression pattern #1 involved in growth cessation and bud formation, and genes showing expression pattern #2 more likely involved in endodormancy maintenance. Another common trend concerning *DAM* genes responsiveness to environmental cues was observed after dormancy establishment, when twigs were exposed to prolonged cold temperatures in controlled conditions. This treatment led to the down-regulation of *DAM* genes in a cultivar-dependent manner (i.e., cultivars with low CR repressed these genes earlier than cultivars with high CR) (Jiménez et al., 2010b; Sasaki et al., 2011; Yamane et al., 2011a; Porto et al., 2016). The reduction of *DAM* gene expression levels by prolonged cold temperatures might be part of a mechanism to control dormancy-release. Indeed, *SVL* regulates budbreak in hybrid aspen by antagonizing GA and ABA pathways (Singh et al., 2018), and its mRNA expression levels are negatively regulated by low temperatures. Still, further studies are necessary to clarify whether *DAM* genes from fruit tree species act as repressors of dormancy-release and/or budbreak.

Finally, *DAM* genes displaying the expression pattern #3 presented highest expression levels before budbreak, usually in the early spring. This pattern was find for apple and kiwifruit genes (Wu et al., 2012, 2017a). Interestingly, two genes showing this pattern of expression (*MdoDAMb* and *AdeSVP2*) were already functionally characterized (see the section “*DAM* and *SVP*-Like Genes Might Act as Growth Inhibitors in Trees”; Wu et al., 2017a,b), suggesting a putative role related to the maintenance of growth suppression upon dormancy establishment, preventing premature growth before budbreak.

## What Regulates the Transcription of *DAM* Genes?

### A Possible Transcriptional Control of *DAM* Genes by the CBF Transcription Factors

**FIGURE 4:**
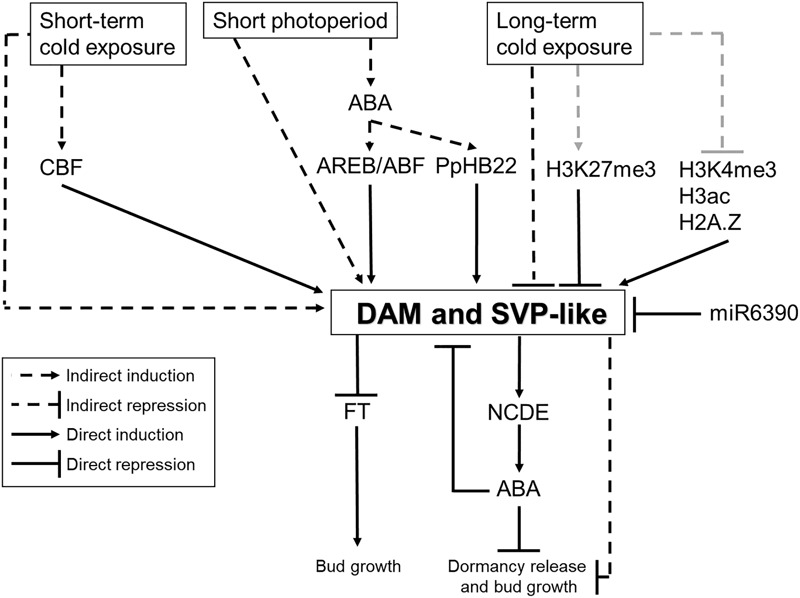
A possible model of molecular control of bud dormancy in temperate fruit tree species mediated by *DAM* and *SVP*-like genes. It has been shown that *DAM* and *SVP*-like genes are regulated by photoperiod and temperature. This regulation is probably mediated by several transcription factors, miRNAs, plant hormones and chromatin modifiers. In turn, DAM and SVP-like transcription factors are involved in the repression of bud growth, probably by affecting the expression of *FT*-like genes and ABA levels. Black lines indicate molecular connections based on previous reports. Gray lines indicate expected genetic interactions that have not been proved yet.

As shown above, the changes in *DAMs* gene expression are transcriptionally modulated by environmental cues. Potential regulators of *DAMs* transcription in response to environmental factors and especially to cold could be a group of transcription factors called dehydration-responsive element-binding (DREB) protein/C-repeat binding factors (CBFs) (reviewed in Akhtar et al., 2012; Zhao et al., 2015) which have been described (Figure 4) as governing cold signaling. Indeed, the expression of these genes is rapidly induced in response to cold in order to increase plant tolerance to freezing stress (Akhtar et al., 2012; Zhao et al., 2015). In agreement with this function, the expression of some peach and Japanese apricot *CBFs* was shown to be induced by cold treatments (Wisniewski et al., 2015; Zhao et al., 2018c). Furthermore, the transcriptional profile of some *CBF* had a maximum peak of expression during winter in Japanese pear (Mimida et al., 2015; Niu et al., 2016) and Japanese apricot (Zhao et al., 2018b).

Characteristically, CBF transcription factors recognize and bind to the CRT/DRE (C-repeat/dehydration responsive element) DNA regulatory motif in the promoters of cold-responsive genes (Zhao et al., 2015). The CRT/DRE motif were found in the promoter region of *DAM* genes in leafy spurge (Horvath et al., 2010), Japanese pear (Saito et al., 2015; Niu et al., 2016), apple (Mimida et al., 2015; Wisniewski et al., 2015) and Japanese apricot (Zhao et al., 2018b). Furthermore, the promoters of some *DAM* genes contain EVENING elements (Horvath et al., 2010), which are known to play a role in circadian regulation and cold induction mediated by CBF (Mikkelsen and Thomashow, 2009). These facts suggest a direct role of CBF transcription factor in controlling the expression levels of *DAM* genes mediated by cold. The experimental confirmation for the binding of CBF transcription factors to promoter regions of *DAM* genes was reported for various temperate tree species. For example, yeast-one-hybrid (Y1H) experiments showed that CBF proteins interact with promoter regions containing CRT/DRE motifs of *PpyDAM1* and *PpyDAM3* in Japanese pear (Niu et al., 2016), and *PmuDAM6* in Japanese apricot (Zhao et al., 2018b). Moreover, Japanese pear CBF transcription factors were able to induce the expression of *PpyDAM1*-*1* and *PpyMADS13*-*3* genes in transient reporter assays (Saito et al., 2015; Niu et al., 2016). The ectopic expression of a peach CBF (*PpeCBF1*) in the apple rootstock variety M26 affected the expression levels of *MdoDAM1* and *MdoDAM3* (called *MdoSVPb* in this review) in buds (Wisniewski et al., 2015). These transgenic trees showed a number of interesting phenotypes such as increased cold hardiness, early growth cessation and leaf senescence, delayed budbreak, growth inhibition, and increased sensitivity to short photoperiod with respect to the onset of dormancy (Wisniewski et al., 2011, 2015).

These data suggest that CBF could participate in the cold-mediated transcriptional activation of *DAM* genes during endodormancy induction. Although this could be related to a mechanism of bud hardiness acquisition, it is reminiscent to the mechanism of flowering inhibition by intermittent-cold sensing that was proposed for Arabidopsis (Seo et al., 2009). In Arabidopsis, CBF transcription factors induce the expression of *FLOWERING LOCUS C* (*FLC*) in response to short periods of cold. Then, FLC acts as floral repressor and prevents flowering to occur under unfavorable conditions (Seo et al., 2009). The intermittent-cold sensing system differs from the vernalization process described in Arabidopsis and other Brassicaceae, where long exposure to cold triggers the epigenetic-mediated inhibition of *FLC* mRNA toward the end of winter, and allows flowering to happen in spring (Michaels and Amasino, 1999; Romera-Branchat et al., 2014). In a similar manner, it has been hypothesized that two mechanisms of cold sensing and signaling could operate during temperate tree dormancy cycle to control *DAM* expression (Horvath, 2009). One mechanism mediated by CBF would operate in direct response to cold at the beginning of winter to activate the transcription of some *DAM* genes, whereas an independent mechanism might regulate the gradual silencing of *DAM* genes upon exposure to low temperature.

### Epigenetic Mechanisms Regulating *DAM* Genes

Recently, a significant amount of publications has specifically addressed how epigenetic mechanisms contribute to the regulation of complex traits including vernalization and bud dormancy (Ríos et al., 2014; Richards et al., 2017). During the winter vernalization in Arabidopsis, histone modifications related to active transcription such as histone H3 at lysine 4 (H3K4me3) are removed from the *FLC* locus (He et al., 2004). Instead, the *FLC* locus is decorated with trimethylation of histone H3 at K27 (H3K27me3) (Bastow et al., 2004), a mark associated with inactive transcription, by the action of the Polycomb Repressive Complex 2 (PRC2) (Whittaker and Dean, 2017). Histone modifications, changes in DNA methylation patterns, and the regulation imposed by small non-coding RNAs (siRNAs) were already observed during dormancy in several perennials (Leida et al., 2012; Zhang et al., 2012; de la Fuente et al., 2015; Saito et al., 2015; Tuan et al., 2016; Conde et al., 2017a,b; Guo et al., 2017; Rothkegel et al., 2017). However, just a few reports have directly analyzed the epigenetic mechanisms acting over *DAM* genes (Figure 4).

Epigenetic regulation of *DAM* genes was first suggested by analyzing dormant buds of leafy spurge (Horvath et al., 2010). During the dormancy cycle, a decrease in the H3K4me3 pattern concomitant to an increase of H3K27me3 was identified in two regions downstream of the transcription start site of *EesDAM1* (Horvath et al., 2010). Interestingly, these changes in epigenetic marks were associated with the downregulation of *EesDAM1* gene during the transition from endodormancy to ecodormancy (Figure 3). This suggests that the seasonality of *DAM* expression in leafy spurge may be controlled in a manner that resembles the *FLC* repression during vernalization.

Further evidence of epigenetic regulation over *DAM* genes was provided in peach. Similar chromatin modifications observed for *EesDAM1* were identified for *PpeDAM6* in two contrasting CR peach cultivars during dormancy transition, i.e., decrease of H3K4me3 and increase of H3K27me3 near the promoter, the translation start site and the largest intron (Leida et al., 2012). Moreover, a decrease in the acetylation levels of H3 (H3ac) near dormancy release, an epigenetic mark that is related to activation of transcription, was shown. A significant enrichment of H3K27me3 was also revealed at specific regions of this locus during dormancy release (de la Fuente et al., 2015), which could contribute to the differential transcription observed for *PpeDAM* genes during dormancy (Figure 3).

In sweet cherry, higher levels of DNA methylation were found in the promoters of *PavMADS1* and *PavMADS2* after CR completion (Rothkegel et al., 2017). An increase in the abundance of small interfering RNAs (siRNA) was associated with the observed *de novo* DNA methylation in the promoter region of *PavMADS1* (Rothkegel et al., 2017). DNA methylation and siRNAs are related to transcription repression when present at the promoter region, which suggests that these epigenetic changes modulate the down-regulation of *DAM* genes during dormancy.

In Japanese pear, the analysis of the chromatin status of *PpyMADS13-1* identified a reduction of H3K4me3 prior to endodormancy release, but no differences were found to H3K27me3 during the dormancy cycle (Saito et al., 2015). Additionally, the authors analyzed the deposition of the histone variant H2A.Z, which in Arabidopsis is responsible to regulate gene expression according to ambient temperature (Kumar and Wigge, 2010). *PpyMADS13-1* chromatin showed a tendency to lose H2A.Z during endodormancy release (Saito et al., 2015), and together with the concomitant down-regulation of this gene at this time point (Figure 3), it indicates that this histone variant may have a negative role over dormancy release.

### Regulatory Intronic Regions Could Be Relevant for the Transcriptional Control of *DAM* Genes

One particular feature of some MADS-box genes is the presence of a long multi-thousand base intron, which usually contains several regulatory sequences that help in the control of gene expression. Several examples demonstrated how regulatory intronic regions control the expression of MADS-box genes such as *AGAMOUS* (*AG*) (Sieburth and Meyerowitz, 1997; Hong, 2003), *AGAMOUS-LIKE 6* (*AGL6*) (Schauer et al., 2009), *FLC* (Gazzani et al., 2003; Liu et al., 2004; Heo and Sung, 2011), *FLOWERING LOCUS M* (*FLM*) (Lutz et al., 2015), among others. Especially for *FLC*, transposon insertions into its largest intron were shown to be responsible for the reduction of *FLC* expression in the early flowering *Landsberg erecta* accession (Gazzani et al., 2003; Liu et al., 2004). In peach, transposon-related insertions in the largest intron of *PpeDAM5* and *PpeDAM6* were observed in low CR cultivars (Yamane et al., 2011c; Zhebentyayeva et al., 2014), resembling *FLC* gene regulation. However, several Japanese pear genotypes with distinct CRs were screened for insertions in the largest intron of *PpyMADS13-1*, but no relationship with the dormancy cycle was found (Saito et al., 2013). Finally, a highly conserved sequence of nearly 200 bp was identified inside the largest intron of apple, Japanese apricot, peach and Japanese pear *DAM* genes, and the sequence conservation was even higher than for some *DAM* exonic sequences (Porto et al., 2016). The evolutionary maintenance of this intronic region suggests a functional role in the transcriptional regulation of *DAM* genes. Whether this region is required for the seasonal mRNA expression profile of *DAM* genes needs further studies.

## What Do We Know About the Biological Function of *DAM* and *SVP*-Like Genes?

### Functional Studies of *DAM* and *SVP*-Like Genes of Fruit Tree Species in Model Plants

SVP is a main factor for Arabidopsis development. The loss-of-function and ectopic expression of *SVP* conferred early and late flowering, respectively (Hartmann et al., 2000; Lee et al., 2007), indicating that SVP functions as a floral repressor during the vegetative phase. In the reproductive phase, SVP regulates the pattern of floral development together with AGL24 and APETALA1 (AP1) (Gregis et al., 2006). Several reports describing the biological function of *DAM* and *SVP*-like genes in perennial species evaluated their function in flowering time and organ development in heterologous systems. The ectopic expression of four kiwifruit *SVP*-like genes from the *35S* promoter in Arabidopsis plants led to abnormalities in inflorescences and floral structures, suggesting a similar role in reproductive development as the Arabidopsis SVP counterpart (Wu et al., 2012). However, only *AdeSVP1* and *AdeSVP3* were able to delay flowering in Arabidopsis and complement the *SVP* loss-of-function. None of the *SVP*-like genes complemented the *agl24* mutant, which shows late flowering (Michaels et al., 2003), indicating that these genes share functional features with *SVP* but not with *AGL24* (Wu et al., 2012). In Japanese apricot, the constitutive expression of *PmuSVP1* and *PmuSVP2* in Arabidopsis caused floral abnormalities, but only *PmuSVP1* delayed flowering (Li et al., 2017). Ectopic expression of *EesDAM1* from leafy spurge in Arabidopsis plants slightly delayed flowering (Horvath et al., 2010).

The results presented above indicate that the misexpression of *SVP*-like genes in Arabidopsis causes similar phenotypes to those produced when *SVP* function is affected (i.e., shifting flowering time and causing floral development abnormalities). Therefore, it is possible to hypothesize that *SVP*-like genes have a molecular function similar to the one of the Arabidopsis *SVP*. However, they have obvious distinct roles in annual and tree species, i.e., control of floral induction and involvement in dormancy cycle, respectively, indicating a strong functional diversification of these genes during the evolution.

### *DAM* and *SVP*-Like Genes Might Act as Growth Inhibitors in Trees

Ectopic expression of *DAM* and *SVP*-like genes was shown to have a growth inhibitory effect able to alter the dormancy cycle in comparison to non-transgenic plants (Figure 4). Different *DAM* and *SVP*-like genes may play distinct roles during different dormancy cycle phases. The constitutive expression of *PmuDAM6* (expression pattern #2) in poplar induced growth cessation, bud set and bud endodormancy (Sasaki et al., 2011). From this result, it is possible to suggest that this gene, and probably other *DAM* genes having expression pattern #2, play a critical function in endodormancy regulation. On the other hand, a weaker effect was observed in poplar trees expressing *PmuDAM1*, which seemed to promote and inhibit apical growth depending on the growth conditions (Yamane and Tao, 2015). In kiwifruit, the overexpression of *AdeSVP2* delayed budbreak of lateral floral buds in the high-chill cultivar *A*. *deliciosa*, but did not affect growth cessation, leaf drop, timing of bud-set and bud formation (Wu et al., 2017b). The delay in budbreak could be overcome by exposing these transgenic vines to long periods of cold. Moreover, the *AdeSVP2* overexpression had no effect on a low chilling kiwifruit species (*A*. *eriantha*). In the light of these results, the role of *AdeSVP2* appears unrelated to cold-mediated dormancy release, but its function could be to prevent premature growth (Wu et al., 2017b). This growth inhibitory function was also observed in transgenic tobacco plants ectopically expressing *AdeSVP2* (Wu et al., 2017b). In apple, transgenic trees overexpressing *MdoDAMb* and *MdoSVPa* showed delayed budbreak without displaying any additional phenotype related to leaf senescence and bud set (Wu et al., 2017a). With the exception of *PmuDAM6*, the other *DAM* and *SVP*-like genes functionally characterized so far seem to be involved in repressing growth after dormancy establishment and/or budbreak after dormancy release rather than promoting bud set and endodormancy.

### *DAM* and *SVP*-Like Genes Might Antagonize *FT*-Like Functions

MADS-box proteins are commonly involved in the control of developmental switches such as floral transition (Andrés and Coupland, 2012) and the specification of floral organ identity (Theißen et al., 2016). In Arabidopsis, SVP regulates the reproductive development mainly by acting as a transcriptional repressor in distinct flowering and hormonal-signaling pathways (Gregis et al., 2013). Notably, it delays flowering by the direct repression of the floral integrators *FT* and *SUPPRESSOR OF OVEREXPRESSION OF CONSTANS* (*SOC1*) under non-inductive conditions (Li et al., 2008; Jang et al., 2009). The protein encoded by *FT* shares high homology with mammalian phosphatidylethanolamine-binding proteins (PEBPs) (Kardailsky et al., 1999; Kobayashi et al., 1999), and in many plant species it functions as a main component of the long-distance signal that induces flowering (and therefore named “florigen”) (Corbeer et al., 2007; Jaeger and Wigge, 2007; Mathieu et al., 2007; Tamaki et al., 2007). The ectopic overexpression of orthologs of *FT* (*FT*-like genes) from several temperate fruit species such as apple, peach, sweet cherry, Japanese apricot and kiwifruit accelerates flowering in Arabidopsis (Kotoda et al., 2010; Tränkner et al., 2010; Varkonyi-Gasic et al., 2013; Yarur et al., 2016). This suggests a conserved function of this gene across taxes (Pin and Nilsson, 2012).

The function of *FT*-like genes has been suggested to be linked to bud dormancy in temperate trees. For instance, the misexpression of poplar *FT* homologs significantly alters growth cessation, bud set and dormancy entrance (Böhlenius et al., 2006; Hsu et al., 2011). Moreover, the ectopic expression of *FT* from *Populus trichocarpa* (*PtrFT1*) in plum also causes premature flowering and overrides the CR for dormancy release (Srinivasan et al., 2012). Similar phenotypes were observed in apple trees overexpressing *MdoFT1* (Tränkner et al., 2010), an apple *FT*-like gene that is found within a QTL related to budbreak date (Allard et al., 2016). In all these species, it is likely that *FT*-like genes antagonize the function of some *DAM* and *SVP*-like genes. Because SVP inhibits *FT* mRNA expression in Arabidopsis, it could be possible that DAM and SVP-like proteins act as transcription repressors of *FT*-like genes in perennial species (Figure 4). In agreement with this hypothesis, Hao et al. (2015) showed by chromatin immunoprecipitation followed by qPCR (ChIP-qPCR) that DAM proteins bind to CArG boxes located in the promoter regions of leafy spurge *FT* genes during endodormancy. Similarly, *in vitro* experiments evidenced that the Japanese pear PpyDAM1 inhibits the expression of *PpyFT2* by binding to its promoter (Niu et al., 2016). Consistently, *PpyFT2* expression levels are opposite to those observed for *PpyDAMs*, remaining low during dormancy and increasing after dormancy release (Ito et al., 2016; Niu et al., 2016). A comparable pattern of expression was reported in apple, in which the expression levels of *MdoFT1* and *MdoFT2* are down-regulated during the winter, and *MdoFT2* is highly upregulated after dormancy release (Kotoda et al., 2010). In hybrid aspen, SVL directly repressed the mRNA expression of *FT1* and ChIP-qPCR experiments showed that Myc-SVL binds to a CArG box on the *FT1* promoter (Singh et al., 2018). Controversially, other studies do not support the direct regulation of *FT*-like genes by DAM and SVP-like transcription factors. Indeed, *FT*-like genes were not found as direct targets of AchSVP2 (Wu et al., 2017c) and PpyMADS13-1 (Saito et al., 2015) in ChIP-seq and transient reporter assays, respectively. However, whether technical issues masked the interaction of the transcription factors with *FT*-like loci, or whether the absence of binding is biologically meaningful requires further studies.

### *DAM, SVP*-Like Genes and Plant Hormones

The involvement of plant hormones in the control of dormancy cycle has been suggested and reviewed in Powell (1987) and Cooke et al. (2012). In particular, it has been suggested that ABA is a growth-inhibiting hormone (Wareing, 1978) that plays a significant role as a regulator of dormancy in seeds (Wareing, 1978; Kermode, 2005; Finkelstein, 2013), and in the bud dormancy cycle (Chmielewski et al., 2017; Tuan et al., 2017; Yue et al., 2017; Tylewicz et al., 2018). This idea is mainly supported by the observation that, in many tree species, ABA content (directly measured or indirectly inferred by gene expression studies) increases within the bud after growth cessation correlating with dormancy induction (Rinne et al., 1994; Karlberg et al., 2010; Chmielewski et al., 2017). However, the above-mentioned studies did not provide genetic evidence on the role of ABA in bud dormancy control. In *Populus* species, an elegant model for ABA-mediated regulation of dormancy in response to photoperiod has been recently proposed (Tylewicz et al., 2018). Short photoperiods induce the accumulation of ABA as well as the expression of genes related to ABA biosynthesis (*ABA DEFICIENT 1* [*ABA1*], *ABA2*, and *9-cis-epoxycarotenoid dioxygenase* [*NCED*]) and signaling (*PROTEIN PHOSPHATASE 2C* [*PP2C*] and *ABA-RESPONSIVE ELEMENT BINDING PROTEIN* [*AREB*/*ABF*]) (Rohde et al., 2002; Ruttink et al., 2007; Karlberg et al., 2010). In turn, the accumulation of ABA in the buds triggers the plasmodesmata closure, which ensures the growth arrest and bud dormancy until sufficient chilling is accumulated (Tylewicz et al., 2018). Expression of the dominant negative *abi1-1* allele of *ABSCISIC ACID-INSENSITIVE3* (*ABI3*) in transgenic poplar trees led to reduced ABA responses. These transgenic trees failed to induce plasmodesmata closure at dormancy onset and display a shorter dormancy cycle compare to wild-types (Tylewicz et al., 2018). Thus, the accumulation of high ABA levels in the buds seems to be crucial for dormancy regulation.

In temperate fruit trees, *DAM* and *SVP*-like genes likely participate in the control of ABA homeostasis (Figure 4). For example, SVL was suggested to inhibit budbreak in hybrid aspen at least partially by the transcriptional activation of genes encoding *NCED3* and ABA receptors (Singh et al., 2018). Similar to the model proposed for poplar, in temperate fruit trees ABA levels are high in dormant buds and decrease during the transition from endo to ecodormancy (Chmielewski et al., 2017). This kinetics of ABA accumulation is related to the activity of *NCEDs* genes in Japanese pear and peach (Wang et al., 2015; Tuan et al., 2017). In Japanese pear, *PpyNCED3* increased toward endodormancy release in lateral flower buds of ‘Kosui’ pear (Tuan et al., 2017). By making use of transient assays with dual luciferase reporter system (LUC assay) and electrophoretic mobility shift assay (EMSA), Tuan et al. (2017) reported that *PpyDAM1* activates the transcription of *PpyNCED3* by binding to a CArG box located in the *PpyNCED3* promoter (Tuan et al., 2017). Therefore, PpyDAM1 would positively regulate the accumulation of ABA during the endodormancy. Interestingly, high levels of ABA could promote the downregulation of *PpyDAM1* during endodormancy release, as a part of a feedback regulatory system (Figure 4) (Tuan et al., 2017). The idea that DAM and SVP-like transcription factors could act in the dormancy cycle through the regulation of ABA homeostasis and signaling was also suggested in other species. For example, the overexpression of the kiwifruit *AdeSVP2* gene may have mimicked the effect of ABA on the plant dehydration response during bud dormancy (Wu et al., 2017b). Indeed, a ChIP-seq experiment showed that the kiwifruit AchSVP2 is able to bind several genes related to ABA, dehydration and osmotic response. However, the AchSVP2 protein did not bind to kiwifruit *NCED* homologs as shown for PpyDAM1 in Japanese pear, and how AchSVP2 would regulate ABA-related responses remains unclear. This suggests the existence of divergent evolutionary mechanisms of ABA regulation of bud dormancy mediated by DAM and SVP-proteins (Wu et al., 2017c).

It is worthy to mention here that, besides ABA, other plant hormones (e.g., GA, cytokinins) are likely involved in the regulation of bud dormancy cycle (see reviews: Tamaki et al., 2007; Cooke et al., 2012; Horvath et al., 2003). Among them, GA could play a role in dormancy release and budbreak (Rinne et al., 2011; Singh et al., 2018), and its biosynthetic pathway was shown to be controlled by SVP-like encoding genes. In Arabidopsis, SVP prevented the accumulation of GA in the SAM during the floral transition by repressing the expression of *GIBBERELLIN 20 OXIDASE 2* (*GA20ox2*), a gene encoding an enzyme required for biosynthesis of GA (Andrés et al., 2014). Similarly, SVL repressed the expression of *GA20ox* genes in hybrid aspen (Singh et al., 2018), suggesting a role of *SVP*-like genes in controlling budbreak. However, whether *DAM* and *SVP*-like genes act on dormancy cycle in fruit tree species by controlling GA levels has not been studied yet.

### Transcriptional Complexes Could Be Involved in Bud Dormancy Control

MADS-box transcription factors form multimeric complexes to regulate floral organ identity (Schwarz-Sommer et al., 1990; Egea-Cortines et al., 1999; Honma and Goto, 2001). The combination of different MADS-box proteins in transcriptional complexes define their function by conferring target specificity. Thus, the same MADS-box protein could have different roles depending on the composition of the complex. Arabidopsis SVP forms complexes with several other MADS-box proteins to regulate floral development and flowering time (de Folter et al., 2005; Gregis et al., 2006; Lee et al., 2007; Balanzà et al., 2014; Mateos et al., 2015). SVP also interacts with FLC to form a complex that inhibits flowering, partially by repression of the floral integrator genes *FT* and *SOC1* (Lee et al., 2007) as well as GA-related genes (Andrés et al., 2014; Mateos et al., 2015). A gene similar to *FLC* (*FLC*-like) has been identified in two independent transcriptomic studies as a putative regulator of apple bud dormancy (Porto et al., 2015; Kumar et al., 2016). However, whether FLC-like proteins form part of a transcriptional complex with DAM and SVP-like to control bud dormancy remains totally unknown. Other protein complexes between DAM and SVP-like and other MADS-box proteins have been recently reported. In Japanese apricot, yeast-two-hybrid (Y2H) and bimolecular complementation assays (BiFC) showed that PmuDAM1, PmuDAM5, and PmuDAM6 could form combinatorial proteins complexes (Zhao et al., 2018b). According to their pattern of expression, the authors argued that these complexes could act during different phases of the dormancy cycle, although these phases were not clearly determined in this study. In addition, an Y2H screening identified an interaction between PmuDAM6 and PmuSOC1 (Kitamura et al., 2016).

The above-mentioned examples illustrate the existence of DAM and SVP-like-containing transcriptional complexes in fruit trees. The composition of these complexes might encrypt particular functions during the dormancy cycle. Thus, unraveling the nature of these complexes, as it was already done in Arabidopsis (de Folter et al., 2005), will help to better understand the function of DAM and SVP-like proteins.

## Final Remarks and Perspectives

There is a significant correlation between the expression profile of *DAM* and *SVP*-like genes and the progression of the dormancy cycle. In addition, the ectopic expression of *DAM* and *SVP*-like genes from strong constitutive promoters (i.e., the *35S* promoter) affected the patterns of dormancy and flowering in a diverse number of perennial plant species. All the studies summarized in this review have enormously contributed to progressively decipher the functions of *DAM* and *SVP*-like genes in temperate fruit tree species. However, with the exception of the *evg* peach mutant and the functional characterization of *SVL* in hybrid aspen, definitive arguments supporting the individual and collective (as part of transcriptional complexes) function of the distinct *DAM* and *SVP*-like genes in dormancy cycle control of these species are still missing. In the era of the genome editing technology, a systematic survey of fruit tree knockout and knockdown mutants are expected to unambiguously characterize their function. In addition, the use of genome-wide technologies as for example ChIP-seq will shed light on the molecular function of this interesting group of genes.

## Author Contributions

VF, EC, and FA contributed to the conception and the writing of the manuscript. BG performed the phylogenetic analysis. All authors contributed to manuscript revision, read and approved the submitted version.

## Conflict of Interest Statement

The authors declare that the research was conducted in the absence of any commercial or financial relationships that could be construed as a potential conflict of interest.
